# He shoots, he scores: Navigating the complex world of transcriptomic scores in the age of artificial intelligence

**DOI:** 10.1002/hem3.53

**Published:** 2024-03-28

**Authors:** David G. Kent

**Affiliations:** ^1^ Department of Biology, York Biomedical Research Institute University of York York UK

The field of artificial intelligence (AI) has captured the world's imagination and the hematology field has been no exception. From digital pathology to sophisticated prognostic algorithms, the exploration of AI tools has dominated the programs of numerous major international conferences and has been splayed across the pages of many hematology journals. The application of these tools to histological images, radiographs, and genomic data has led the way, likely due to their dominance in the clinical diagnostics arena. Lagging behind from a clinical integration perspective, however, is the integration of data sets such as transcriptomics and flow cytometry, where AI tools appear to be deployed in a more experimental/liberal manner to generate hypotheses with largely experimental goals in mind. This has led to the emergence of transcriptomic signatures with highly variable levels of applicability and robustness, and the challenge has become knowing how to identify the good signatures from the bad ones when it seems that just about any data set can be used to generate a “score.”

In trying to navigate this space, the importance of validation data sets cannot be underestimated. While this is particularly true of transcriptional scores that are intended for clinical use, the principles are equally important for their application in experimental biology. In practice, researchers can be tempted to assign greater credibility to scores emerging from a particular research group or scores that have been picked up in the literature frequently, but in reality, this does not always translate to identifying the most useful transcriptional signatures. Testing signatures against known benchmarks are much more robust but often involves adding additional time or experimentation to the selection process. For example, in the world of hematopoietic stem cell biology, an “HSC Score” should be accompanied by transplantations to demonstrate that cells expressing these markers are indeed functional stem cells that read out positively in transplantation assays—something that is regularly absent from research papers.^1^

As an example, we recently showed that transcriptomic scores developed in one system (in our case, expanded mouse hematopoietic stem cells) can be readily applicable to other systems (e.g., human hematopoietic stem and progenitor cells), but this transfer across systems was highly reliant on direct linkage of the transcriptomic scoring to functional outcomes of those cells across multiple cellular states.^2^ While this is relatively easy to do across extremely well‐characterized mouse hematopoietic stem cell systems (inbred strains, robust single‐cell functional assays across numerous labs), the challenges presented by human data are far greater and, as such, most studies have been restricted to developing static transcriptomic profiling of a particular cell type across highly heterogeneous patient samples. These types of data sets can often be reproduced in the same center with the same machines and protocols but are less commonly useful across other research data sets.

To identify reliable transcriptomic patterns across patient cohorts, much larger data sets are therefore required and this is the approach recently taken by Li et al. who used transcriptomic data from 351 patients to try and improve patient stratification of pediatric lysine methyltransferase 2A‐rearranged acute myeloid leukemia (KMT2A‐r AML).^3^ Using Least Absolute Shrinkage and Selection Operator modelling, they were able to identify a seven‐gene transcriptional signature which they dubbed a pKMT2A7 score. A high pKMT2A7 score was then shown in a validation cohort to be an independent risk factor for both overall survival and event‐free survival when combined with age and MLL3 mutational status. The pKMT2A7 score was particularly useful in identifying low‐risk patients. Assembling transcriptomic data sets from such large numbers of patients is not trivial, though, both from an economic and a data analysis point of view.

The above study demonstrates the utility of expanding beyond a single molecular modality to improve patient stratification, but this then also prompts the need to standardize these other modalities for application in a clinical setting. Whereas this has been relatively straightforward in the context of collecting blood cell parameters or genomic information^4^, ^5^ (both of which are far less subject to the vagaries of sample/cell isolation and preparation), integrating transcriptomic, flow cytometry, or cytokine expression levels^6^ presents additional challenges from a standardization point of view.

Once those standardized platforms are in place at more centers across the world, we will be able to take better advantage of new tools in artificial intelligence to analyze these data and develop stronger algorithms for risk stratification. Until then, however, we urgently need to work across experimental, clinical, and computational communities to define sample preparation and cell isolation standards for transcriptomic, flow cytometry, and cytokine/chemokine platforms that reach beyond the cataloging of data and push toward ensuring that the data generated represent the right molecules from the right cells at the right time. Garbage in, garbage out—the need for data hygiene is enormous.

## AUTHOR CONTRIBUTION

David G. Kent is the sole author and wrote the article.

## CONFLICT OF INTEREST STATEMENT

The author declares no relevant conflict of interest.

## Data Availability

Data sharing not applicable to this article as no datasets were generated or analysed during the current study.

## References

[1] Jassinskaja M , Gonka M , Kent DG . Resolving the hematopoietic stem cell state by linking functional and molecular assays. Blood. 2023;142:543‐552. 10.1182/blood.2022017864 36735913 PMC10644060

[2] Che JLC , Bode D , Kucinski I , et al. Identification and characterization of in vitro expanded hematopoietic stem cells. EMBO Rep. 2022;23:e55502. 10.15252/embr.202255502 35971894 PMC9535767

[3] Li J , Zong S , Wan Y , et al. Integration of transcriptomic features to improve prognosis prediction of pediatric acute myeloid leukemia with KMT2A rearrangement. HemaSphere. 2023;7:e979. 10.1097/HS9.0000000000000979 38026790 PMC10666994

[4] Papaemmanuil E , Gerstung M , Bullinger L , et al. Genomic classification and prognosis in acute myeloid leukemia. N Engl J Med. 2016;374:2209‐2221. 10.1056/NEJMoa1516192 27276561 PMC4979995

[5] Grinfeld J , Nangalia J , Baxter EJ , et al. Classification and personalized prognosis in myeloproliferative neoplasms. N Engl J Med. 2018;379:1416‐1430. 10.1056/NEJMoa1716614 30304655 PMC7030948

[6] Øbro NF , Grinfeld J , Belmonte M , et al. Longitudinal cytokine profiling identifies GRO‐α and EGF as potential biomarkers of disease progression in essential thrombocythemia. HemaSphere. 2020;4:e371. 10.1097/HS9.0000000000000371 32647796 PMC7306314

